# A Risk Prediction Model for Screening Bacteremic Patients: A Cross Sectional Study

**DOI:** 10.1371/journal.pone.0106765

**Published:** 2014-09-03

**Authors:** Franz Ratzinger, Michel Dedeyan, Matthias Rammerstorfer, Thomas Perkmann, Heinz Burgmann, Athanasios Makristathis, Georg Dorffner, Felix Lötsch, Alexander Blacky, Michael Ramharter

**Affiliations:** 1 Department of Laboratory Medicine, Division of Medical and Chemical Laboratory Diagnostics, Medical University of Vienna, Vienna, Austria; 2 Department of Medicine I, Division of Infectious Diseases and Tropical Medicine, Medical University Vienna, Vienna, Austria; 3 Department of Laboratory Medicine, Division of Clinical Microbiology, Medical University of Vienna, Vienna, Austria; 4 Section for Artificial Intelligence, Center for Medical Statistics, Informatics and Intelligent Systems, Medical University of Vienna, Vienna, Austria; 5 Clinical Institute for Hospital Hygiene, Medical University of Vienna, Vienna, Austria; 6 Institut für Tropenmedizin, Universität Tübingen, Tübingen, Germany; Brighton and Sussex Medical School, United Kingdom

## Abstract

**Background:**

Bacteraemia is a frequent and severe condition with a high mortality rate. Despite profound knowledge about the pre-test probability of bacteraemia, blood culture analysis often results in low rates of pathogen detection and therefore increasing diagnostic costs. To improve the cost-effectiveness of blood culture sampling, we computed a risk prediction model based on highly standardizable variables, with the ultimate goal to identify via an automated decision support tool patients with very low risk for bacteraemia.

**Methods:**

In this retrospective hospital-wide cohort study evaluating 15,985 patients with suspected bacteraemia, 51 variables were assessed for their diagnostic potency. A derivation cohort (n = 14.699) was used for feature and model selection as well as for cut-off specification. Models were established using the A2DE classifier, a supervised Bayesian classifier. Two internally validated models were further evaluated by a validation cohort (n = 1,286).

**Results:**

The proportion of neutrophile leukocytes in differential blood count was the best individual variable to predict bacteraemia (ROC-AUC: 0.694). Applying the A2DE classifier, two models, model 1 (20 variables) and model 2 (10 variables) were established with an area under the receiver operating characteristic curve (ROC-AUC) of 0.767 and 0.759, respectively. In the validation cohort, ROC-AUCs of 0.800 and 0.786 were achieved. Using predefined cut-off points, 16% and 12% of patients were allocated to the low risk group with a negative predictive value of more than 98.8%.

**Conclusion:**

Applying the proposed models, more than ten percent of patients with suspected blood stream infection were identified having minimal risk for bacteraemia. Based on these data the application of this model as an automated decision support tool for physicians is conceivable leading to a potential increase in the cost-effectiveness of blood culture sampling. External prospective validation of the model's generalizability is needed for further appreciation of the usefulness of this tool.

## Background

Bacteraemia is a frequent and severe condition with an annualized incidence of 122 per 100.000 people. The mortality rate ranges between 14% and 37% [1]–[3]. Risk factors for bacteraemia are advanced patient's age, urinary or indwelling vascular catheter, fulfilment of two or more SIRS criteria, impaired renal or liver function, malignancy or other chronic co-morbidities [4]–[8]. Although blood culture analysis is considered the gold standard for diagnosing bacteraemia in patients with suspected blood stream infection, the clinical decision of when to take a blood culture is not trivial. Despite profound knowledge about the pre-test probability of positive blood culture results, which is strongly influenced by the site of infection, true positive rates identifying a causative pathogen are in a low range when consecutively assessed (4.1%–7%) [9]–[11]. Compared to the true positive rate, false positive results due to contamination are in a similar or even in a higher range, varying between 0.6% to over 8% [11]–[13]. Importantly, these imperfections of blood culture analysis have an important economic impact, resulting in a 20% increase of total hospital costs for patients with false positive blood cultures [14]–[17]. Economic analyses estimate the costs related to a single false positive blood culture result between $6,878 and $7,502 per case [17]–[19].

To increase the cost effectiveness of blood culture analysis, the identification of targeted patient cohorts is therefore highly needed. Several prediction systems for bacteraemia in special patient cohorts have been published with ROC-AUCs in a moderate range [20]–[24]. However, physicians are arguably inefficient in applying a multitude of available prediction scores for specific conditions and specific patient cohorts [25], [26]. The aim of the current study was therefore to establish a machine learning based prediction system for inpatients and outpatients with suspected bacteraemia using highly standardized and routinely available laboratory parameters to identify those patients for whom blood culture sampling may safely be omitted due to very low pre-test probability for bacteraemia.

## Material and Methods

### Study Design and Data Collection

The current study was designed as a retrospective cohort study, including inpatients and outpatients at the Vienna General Hospital, Austria, a 2,116-bed tertiary teaching facility. Between January 2006 and December 2010, patients with the clinical suspicion to suffer from bacteraemia were included if blood culture analysis was requested by the responsible physician and blood was sampled for assessment of haematology and biochemistry. Patients younger than 18 years and patients with unavailable laboratory parameter results were excluded. Patients with a potential blood culture contaminant and those with missing or inaccurate identification to the species level were excluded from further analysis. Blood culture contamination was defined according to the criteria of Hall and Lyman [27]. Furthermore, patients with rare blood culture isolates (less than 0.15% frequency of positives) were also excluded. Patients'age, gender and 49 laboratory parameters (see table 1) were used in the analysis. All laboratory parameters had been assessed in accordance to parameter specific SOPs at the Clinical Department of Laboratory Medicine, Medical University Vienna, an ISO 9001:2008 certified and ISO 15189:2008 accredited facility. Anonymous raw data can be request by contacting the corresponding author. Following national regulations each request will be evaluated for approval by the local human data safety commission.

**Table 1:** Patient characteristics and variables analysed.

			All	No Bacteraemia	Bacteraemia		
M1	M2	Variable	N = 15,985	N = 14,699	N = 1,286	p-value	ROC-AUC
X	X	Neutrophiles %	15,181	77.7 (68.7–84.6)	85.8 (78.3–90.5)	<0.0001	0.696
X	X	Lymphocytes (G/L)	15,695	1.1 (0.7–1.6)	0.7 (0.4–1.1)	<0.0001	0.683
		Lymphocytes % (mg/dl)	15,250	11.6 (7.1–18.6)	7 (4.15–12.2)	<0.0001	0.674
X	X	Monocytes %	15,268	8.1 (5.8–10.7)	6.1 (3.5–8.8)	<0.0001	0.645
		BUN (mg/dl)	15,800	16.2 (11.4–25.8)	22.5 (14.7–37.78)	<0.0001	0.633
X		Eosinophils (G/L)	15,373	0.6 (0.1–1.8)	0.2 (0–0.8)	<0.0001	0.641
X	X	Eosinophil %	15,831	0.1 (0–0.2)	0 (0–0.1)	<0.0001	0.626
X	X	Bilirubin (mg/dl)	14,431	0.75 (0.52–1.19)	1.02 (0.66–1.73)	<0.0001	0.621
X	X	Age	15,985	58 (42–69)	65 (53–74)	<0.0001	0.611
X	X	Creatinine (mg/dl)	15,813	0.99 (0.81–1.31)	1.2 (0.89–1.87)	<0.0001	0.611
		Basophiles %	15,375	0.2 (0.1–0.3)	0.1 (0.1–0.2)	<0.0001	0.606
X	X	Sodium (mmol/L)	14,542	138 (135–140)	136 (133–139)	<0.0001	0.602
X		ALP (U/L)	14,479	83 (62–120)	100 (72–164)	<0.0001	0.601
X		GGT (G/L)	14,629	48 (25–112)	73 (35–180)	<0.0001	0.599
X	X	Monocytes (G/L)	15,710	0.8 (0.5–1.1)	0.6 (0.3–1)	<0.0001	0.598
X	X	CRP (mg/dl)	15,820	8.39 (2.77–16.15)	11.68 (5.22–21.19)	<0.0001	0.596
		CHE (kU/L)	13,353	4.66 (3.2–6.29)	3.94 (2.66–5.48)	<0.0001	0.591
X		MG (mmol/L)	13,989	0.81 (0.73–0.89)	0.77 (0.68–0.86)	<0.0001	0.582
		PLT (G/L)	15,940	206 (142–279.25)	180.5 (115–248)	<0.0001	0.575
		RDW (%)	15,924	14.4 (13.3–15.925)	14.9 (13.7–16.6)	<0.0001	0.572
		Normotest (%)	13,339	84 (67–101)	78 (60–94)	<0.0001	0.571
		Albumin (G/L)	14,187	33.7 (28–39.3)	32 (26.925–36.7)	<0.0001	0.568
X		RBC (T/L)	15,478	3.9 (3.4–4.5)	3.7 (3.2–4.2)	<0.0001	0.567
X		Amylase (U/L)	11,783	50 (34–77)	44 (28–70)	<0.0001	0.565
X		Cholesterol (mg/dl)	10,565	146 (114–183)	132 (105–171)	<0.0001	0.564
		Glucoses (mg/dl)	11,350	113 (96–137)	121 (99–154)	<0.0001	0.559
		Haematocrit (%)	15,941	34.4 (29.8–39.2)	33.1 (28.5–37.5)	<0.0001	0.561
		Uric acid (mg/dl)	12,709	5 (3.7–6.5)	5.5 (3.9–7.6)	<0.0001	0.562
		Neutrophiles (G/L)	15,181	7.3 (4.6–10.7)	8.4 (5.23–12.7)	<0.0001	0.559
		ASAT (U/L)	14,745	31 (22–56)	37 (24–70.25)	<0.0001	0.558
X		CK (U/L)	13,763	82 (42–190)	67 (34–142)	<0.0001	0.557
X		Haemoglobin(G/L)	15,942	11.4 (9.9–13.2)	11.1 (9.5–12.6)	<0.0001	0.554
		ALAT (U/L)	14,919	26 (16–47)	30 (18–60)	<0.0001	0.55
		PAMY (U/L)	8228	22 (14–36)	20 (12–34)	0.0001	0.544
		Fibrinogen (mg/dl)	13,211	526 (393–667)	546 (424–701)	0.0001	0.538
X		Phosphate(mmol/L)	14,664	1 (0.81–1.2)	0.95 (0.76–1.19)	<0.0001	0.537
		Calcium (mmol/L)	14,592	2.23 (2.09–2.35)	2.21 (2.08–2.33)	0.0001	0.533
		TP (G/L)	14,301	65.8 (56.8–73.4)	64.7 (56.4–71.5)	0.0019	0.528
		LDH (U/L)	14,150	239 (186–334)	249 (199–331.5)	0.0037	0.527
		MCH (fl)	15,941	29.7 (28.3–30.9)	29.8 (28.5–31.2)	0.0019	0.526
		MCV (pg)	15,941	88.1 (84.6–91.9)	88.6 (84.8–92.5)	0.0044	0.524
		Basophiles (G/L)	15,827	0 (0–0)	0 (0–0)	<0.0001	0.47
		PDW (%)	14,776	12 (10.8–13.4)	12.1 (10.8–13.7)	n.s.	
		aPTT (sec)	13,251	37.8 (34.2–42.8)	37.8 (34.2–43)	n.s.	
		Lipases (U/L)	11,988	23 (13–40)	22 (12–38)	n.s.	
		MCHC (g/dl)	15,941	33.5 (32.6–34.4)	33.6 (32.7–34.5)	n.s.	
		MPV (fl)	15,214	10.3 (9.7–11)	10.4 (9.7–11.1)	n.s.	
		Potassium (mmol/L)	13,774	3.95 (3.67–4.3)	3.97 (3.595–4.365)	n.s.	
		Triglyceride (mg/dl)	10,549	115 (83–164)	118 (85–170)	n.s.	
		WBC (G/L)	15,477	9.58 (6.64–13.46)	10.205 (6.61–14.86)	n.s.	
		Female: Male	15,985	58.06%: 41.94%	58.79%: 41.21%	n.s.	

total study population (n = 15.985); green: selected variables, red: deselected variables; for variable selection the derivation set was used. M1 =  model 1, M2 =  model 2, CRP =  C-reactive protein, ALP =  alkaline phosphatase, CK =  creatinine kinases, GGT =  gamma-glutamyl transpeptidase, MG =  magnesium, RBC =  red blood count, ALAT =  alanine transaminase, ASAT =  aspartate transaminase, BUN =  blood urea nitrogen, CHE =  cholinesterase, LDH =  lactate dehydrogenase, MCH =  mean corpuscular hemoglobin, MCV =  mean corpuscular volume, PAMY =  pancreas amylase, RDW =  red blood cell distribution width, TP =  total protein, PDW =  platelet distribution width, aPTT =  activated partial thromboplastin time, MCHC =  Mean corpuscular hemoglobin concentration, MPV =  mean platelet volume, WBC =  white blood count;

### Ethical Considerations

The study was approved by the local Ethics Committee of the Medical University Vienna (EC-Nr.: 333/2011) and conducted in accordance to the Declaration of Helsinki (1965, including current revisions), the rules of Good Clinical Practice (GCP, European Union) and the standards for the reporting of diagnostic accuracy studies (STARD). Since a retrospective study design was applied, informed consent was not sought from study participants. To assure anonymity, every study participant was assigned a consecutive identification number, which was exclusively used for further analysis.

### Evaluation method

The data set was divided into a derivation set (Jan 1, 2006 to Jul 31, 2010) and a validation set (Aug 1, 2010 to Dec 31, 2010) based on the date of inclusion. For feature selection and model training the derivation set was used. Feature selection and internal validation of the trained model was performed using a 10 fold cross validation scheme. Results of the internal validation were taken to set cut-off points for risk stratification of the study population. The Youden index method was applied to set optimal cut-off points [28], [29]. Using likelihood ratios (LR; LR^−^:0.12, LR^+^:4.93, see figure S1) of corresponding cut-off values, three strata were established to group the patients into a low risk, intermediate risk and high risk group. For the low risk group a cut-off point for the classification probability was set to yield 1% post-test probability for bacteraemia. For the high risk group, a cut-off point resulting in more than 30% post-test probability was predefined. Classification probabilities between these defined cut off points were allocated to the intermediate risk group. To externally validate the discriminatory potency of the previously trained algorithm and risk strata, the validation set was used.

### Statistical Analysis

For statistical analysis, WEKA (Version 3.7.10, GNU General Public License) and R (Version 3.0.2, GNU General Public License) were used [30]. Descriptive statistics of all variables indicated are given as median and interquartile range. For single variable analysis, the Mann-Whitney U-test, Pearson's chi-squared test and area under the receiver operating characteristic curve (ROC-AUC) analysis of individual variables were applied [31]. To train the multivariable models, variables with a high discriminative power were selected, using the wrapper subset evaluator algorithm and the correlation feature selection (CFS) subset evaluator of WEKA. The wrapper approach aims at selecting a relevant set of variables for a specific classification algorithm (in our case the A2DE algorithm, see below) [32]. The CFS subset evaluator evaluates the discriminatory power of a variable subset with respect to their inter-correlation to each other [33]. Furthermore, the effect of each variable was evaluated by a step-wise deletion of variables in the order of their individual Pearson's correlation coefficient with respect to the outcome.

For statistical modelling, several major groups of supervised machine learning algorithms were applied, including Bayesian classifiers such as Naïve Bayes, artificial neural networks such as multilayer perceptrons, or support vector machines. The best results were consistently achieved with the averaged 2-dependence estimators (A2DE) algorithm. The A2DE, belonging to the averaging *n*-dependence estimator classifier group, is a semi-Naïve Bayes method [34]. This group of algorithms assumes that each predicting variable depends on the outcome-class and *n* other variables. In case of the A2DE classifier, *n* equals two, whereas the *classic* Naïve Bayes algorithm is a zero-dependence estimator, assuming that all variables are conditionally independent from each other [35], [36]. In many real-world applications, this independence assumption is violated, leading to inadequate results. The Naïve Bayes algorithm requires a two dimensional table (outcome class and predicting variable) for indexing the probability estimates. In contrast, the A2DE requires two additional dimensions for the estimation of the two additional variable dependencies. Further, these classifiers aggregate the predictions made by a collection of *n*-dependence estimators [37]. These procedures decrease the bias but slightly increase the model's variance [38]. However, comprehensive experimental evaluations indicate that the A2DE's trade-off between bias and variance results in a good predictive accuracy for many applications and data sets [39]–[41].

For ROC-curve comparison, a paired t-test (comparison of paired cross validation folds), the DeLong test or the Hanely and McNeil comparison test were applied to values of the ROC-AUC [42]–. Furthermore, 95% confidence intervals of performance measures, including sensitivity, specificity, negative predictive value (NPV) or positive predictive value (PPV), were calculated with bootstrapping (2,000 iterations) [45]. Where appropriate, the Bonferroni-Holm method was used to control for type I errors, related to multiple testing. Statistical significance was defined as a p-value less than 0.05.

## Results

### Study population

**Figure 1:**
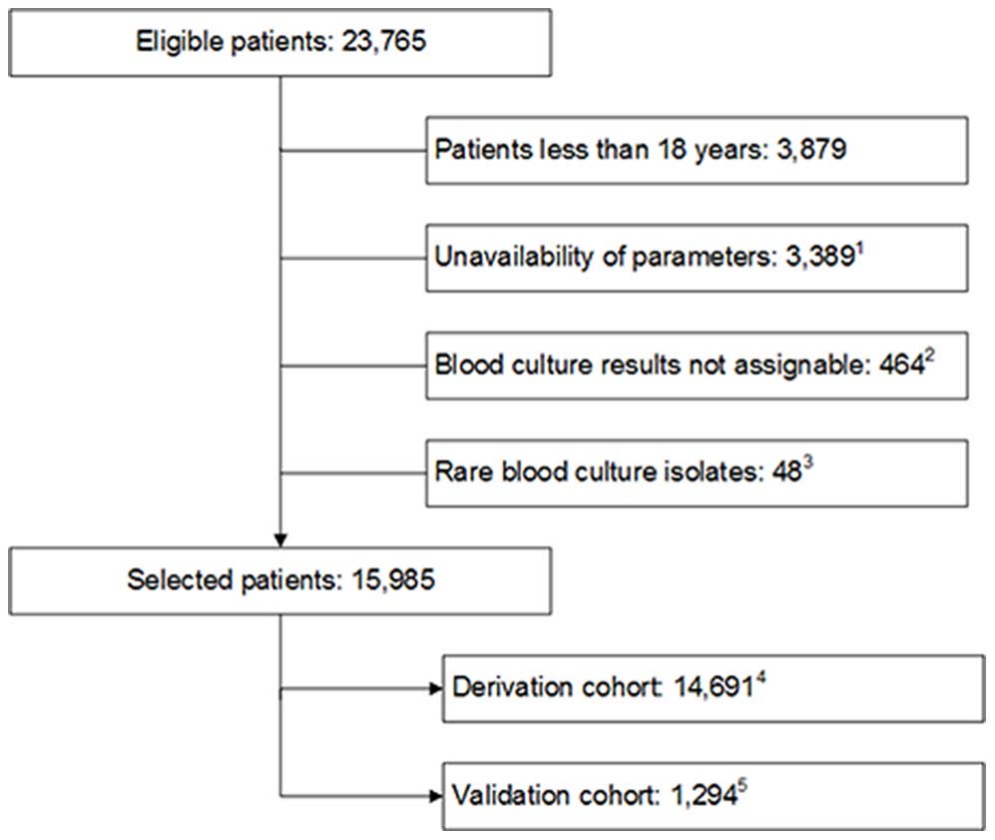
Selection process of the study population. ^1^unavailability of laboratory variables, ^2^Contaminations or fungal growth, ^3^blood culture results with less than 0.001% frequency, ^4^study patients treated between Jan 1, 2006 and Jul 31, 2010, ^5^study patients treated between Aug 1, 2010 and Dec 31, 2010.

Between January 2006 and December 2010, blood culture analysis was requested for 23,765 patients. Figure 1 presents the selection process of patients. Patients less than 18 years old (n = 3,879), patients with unavailable laboratory parameter results (n = 3,389), patients with blood culture contamination, patients with blood culture results having missing or inaccurate identification to the species level and fungal growth (n = 464) and patients with rare blood culture isolates (n = 48) were excluded from analysis. The final study population consisted of 15,985 patients. Among them, 1,286 patients (8%) had a positive blood culture result. Most prevalent bacteria were *E. coli* (n = 406, 31.5%), *S. aureus* (n = 297, 23.1%), and *K. pneumonie* (n = 83, 6.5%). Patient characteristics are presented in Table 1. According to a predefined temporal criterion (cut-off date: Aug 1, 2010), the data set was divided into a derivation set (n = 14,691, 8% bacteraemia) and a validation set (n = 1,294, 8.2% bacteraemia).

### Feature selection and model training

Among 51 available variables in the derivation set, 40 variables resulted in a statistically significant difference between bacteraemia and non-bacteraemia patients. The best individual discriminatory variable was the proportion of neutrophil leukocytes in differential blood count (p<0.0001) with an ROC-AUC of 0.694 (CI: 0.686–0.702). At the Youden Index cut-off point, the relative amount of neutrophils resulted in 61.95% (59.1%–64.7%) sensitivity and 67.6% specificity (66.8%–68.4%), respectively. Among all variables, 20 variables were selected by the wrapper approach (model 1), which were further evaluated by the CFS subset evaluator (model 2). Finally, model 2 consisted of ten variables, including patient's age, proportion of neutrophils, monocytes (absolute and relative value), eosinophils (absolute value), lymphocytes (absolute value), sodium, C-reactive protein, creatinine and total bilirubin (Table 2). Also other feature selection steps were evaluated, resulting in models with lower ROC-AUCs than described below.

**Table 2:** Differences between derivation cohort and validation cohort.

			Derivation cohort		Validation cohort		
M1	M2	Variable	Missing	Median (IQR)	Missing	Median	p-values
X	X	Age	0.0%	58.0 (43.0–70.0)	0.0%	58.0 (41.0–70.0)	0.712
X	X	Creatinine (mg/dl)	1.1%	1.0 (0.8–1.4)	1.0%	1.0 (0.8–1.3)	0.242
X	X	CRP (mg/dl)	1.1%	8.6 (2.9–16.5)	0.8%	9.4 (3.2–17.5)	0.014
X	X	Eosinophil G/L	1.0%	0.10 (0.0–0.10)	1.5%	0.1 (0.0–0.1)	0.602
X	X	Bilirubin (mg/dl)	9.9%	0.8 (0.5–1.2)	8.7%	0.8 (0.5–1.2)	0.369
X	X	Lymphocytes (G/L)	1.8%	1.0 (0.70–1.50)	2.2%	1.0 (0.6–1.5)	0.092
X	X	Monocytes (G/L)	1.7%	0.70 (0.50–1.10)	2.2%	0.8 (0.5–1.1)	0.952
X	X	Monocytes %	4.5%	7.9 (5.7–10.6)	5.3%	7.8 (5.6–10.3)	0.243
X	X	Neutrophiles %	5.0%	78.3 (69.2–85.2)	5.9%	79.1 (69.0–86.3)	0.047
X	X	Sodium (mmol/L)	8.8%	137.0 (135.0–140.0)	12.4%	138.0 (135.0–140.0)	0.041
X		Amylase (U/L)	26.7%	49.0 (33.0–76.0)	22.3%	51.0 (33.0–76.0)	0.434
X		ALP (U/L)	9.6%	84.0 (63.0–122.8)	8.2%	82.0 (63.0–122.0)	0.700
X		Cholesterol (mg/dl)	34.4%	145.0 (113.0–182.0)	29.0%	143.0 (109.0–184.0)	0.323
X		CK (U/L)	14.2%	80.0 (42.0–183.0)	11.0%	84.5 (41.0–214.5)	0.327
X		Eosinophils (G/L)	3.8%	0.60 (0.10–1.70)	4.7%	0.6 (0.1–1.7)	0.816
X		GGT (G/L)	8.6%%	49.0 (25.0–117.0)	7.3%	51.0(26.0–114.0)	0.747
X		Haemoglobin(G/L)	0.3%	11.40 (9.9–13.2)	0.2%	11.2 (9.7–12.9)	<0.001
X		MG (mmol/L)	12.8%	0.81 (0.72–0.89)	9.8%	0.82(0.74–0.91)	<0.001
X		Phosphate(mmol/L)	8.5%	1.0 (0.81–1.20)	6.1%	0.98 (0.80–1.16)	0.007
X		RBC (T/L)	3.2%	3.90 (3.40–4.50)	3.6%	3.9 (3.3–4.4)	0.06

green: selected variables, red: deselected variables; for variable selection the derivation set was used. After application of the Bonferroni-Holm procedure, haemoglobin and magnesium was found to significantly differ between the sets. CRP =  C-reactive protein, ALP =  alkaline phosphatase, CK =  creatinine kinases, GGT =  gamma-glutamyl transpeptidase, MG =  magnesium, RBC =  red blood count;

A number of applicable classes of supervised machine learning techniques including artificial neural networks and support vector machines were screened in the model selection process. Figure S2 presents ROC-curves of various classifiers. The best results in ROC curve analysis were achieved by applying the A2DE classifier yielding an ROC-AUC of 0.767 (CI: 0.754–0.781) in model 1, and of 0.759 (CI: 0.745–0.773) in model 2, respectively. This classifier is conceptually simpler than other algorithms available, and presented constantly better results in ROC-AUC analysis than other classifier tested. Generally, the models'calibration appears to be good. Calibration plots are shown in figure S3. Model 1 shows a modest risk for overestimation for patients at higher bacteraemia risk. This overestimation effect is not seen in model 2, which therefore appears to be very well calibrated.

Using the Youden Index method to set an optimal cut-off point, model 1 yielded 72.1% sensitivity and 70.3% specificity with 17.3% PPV and 96.7% NPV. Model 2 yielded 67.7% sensitivity and 72.8% specificity with 17.8% PPV and 96.7% NPV. Different cut-off points were used to establish a low risk, an intermediate risk and a high risk group for bacteraemia. Table 3 summarizes diagnostic prediction measures when using different cut-off points. Importantly, the low risk group demonstrates a NPV of 98.84 (model 1) and 99.14 (model 2), respectively.

**Table 3:** Results of the models'diagnostic performances at predefined cut-off points.

		Model 1 Youden Index1	Model 1 Low Risk2	Model 1 High Risk3	Model 2 Youden Index1	Model 2 Low Risk2	Model 2 High Risk3
**Derivation cohort**	Sensitivity	72.12 (67.20–78.56)	97.29 (96.19–98.14)	34.66 (31.94–37.45)	67.71 (63.73–72.46)	99.15 (98.45–99.59)	29.66 (27.07–32.36)
	Specificity	70.30 (63.60–74.24)	20.20 (19.52–20.89)	91.45 (90.97–91.92)	72.81 (67.71–75.52)	8.49 (8.02–8.97)	93.88 (93.46–94.28)
	LR+	2.43 (2.32–2.54)	1.22 (1.20–1.23)	4.05 (3.68–4.46)	2.50 (2.38–2.63)	1.08 (1.08–1.09)	4.85 (4.34–5.41)
	LR-	0.42 (0.38–0.46)	0.13 (0.10–0.19)	0.71 (0.69–0.75)	0.46 (0.42–0.49)	0.10 (0.05–0.19)	0.75 (0.72–0.78)
	PPV	17.28 (15.54–18.64)	9.62 (9.10–10.17)	26.15 (23.99–28.41)	17.75 (16.18–18.86)	8.64 (7.60–8.48)	29.74 (27.14–32.44)
	NPV	96.66 (96.23–97.18)	98.84 (98.37–99.21)	94.13 (93.71–94.52)	96.65 (95.91–96.64)	99.14 (98.42–99.58)	93.86 (93.44–94.26)
**Validation cohort**	Sensitivity	79.25 (70.28–86.51)	98.11 (93.35–99.77)	28.30 (19.98–37.88)	80.19 (71.32–87.30)	97.17 (91.95–99.41)	29.25 (20.81–38.87)
	Specivity	68.35 (65.62–70.99)	16.84 (14.75–19.09)	95.03(93.64–96.20)	70.03 (67.34–72.63)	12.96 (11.10–15.01)	94.28 (92.80–95.53)
	LR+	2.50 (2.20–2.85)	1.18 (1.14–1.22)	5.70 (3.85–8.43)	2.68 (2.35–3.04)	1.12 (1.07–1.16)	5.11 (3.51–7.44)
	LR-	0.30 (0.21–0.44)	0.11 (0.03–0.44)	0.75 (0.67–0.85)	0.28 (0.19–0.42)	0.22 (0.07–0.67)	0.75 (0.66–0.85)
	PPV	18.26 (14.84–22.10)	9.52 (7.85–11.42)	33.71 (23.97–44.57)	19.27 (15.70–23.27)	9.06 (7.45–10.88)	31.31 (22.32–41.47)
	NPV	97.36 (96.03–98.34)	99.01 (96.47–99.88)	93.69 (92.17–95.00)	97.54 (96.26–98.47)	98.09 (94.52–99.60)	93.72 (92.20–95.03)

model 1: 20 variables; model 2: 10 variables;

1 Youden Index method,

2 Cut-off at LR^−^ 0.12,

3 Cut-off at LR^+^ 4.93.

### Effects of feature reduction and missing values

**Figure 2:**
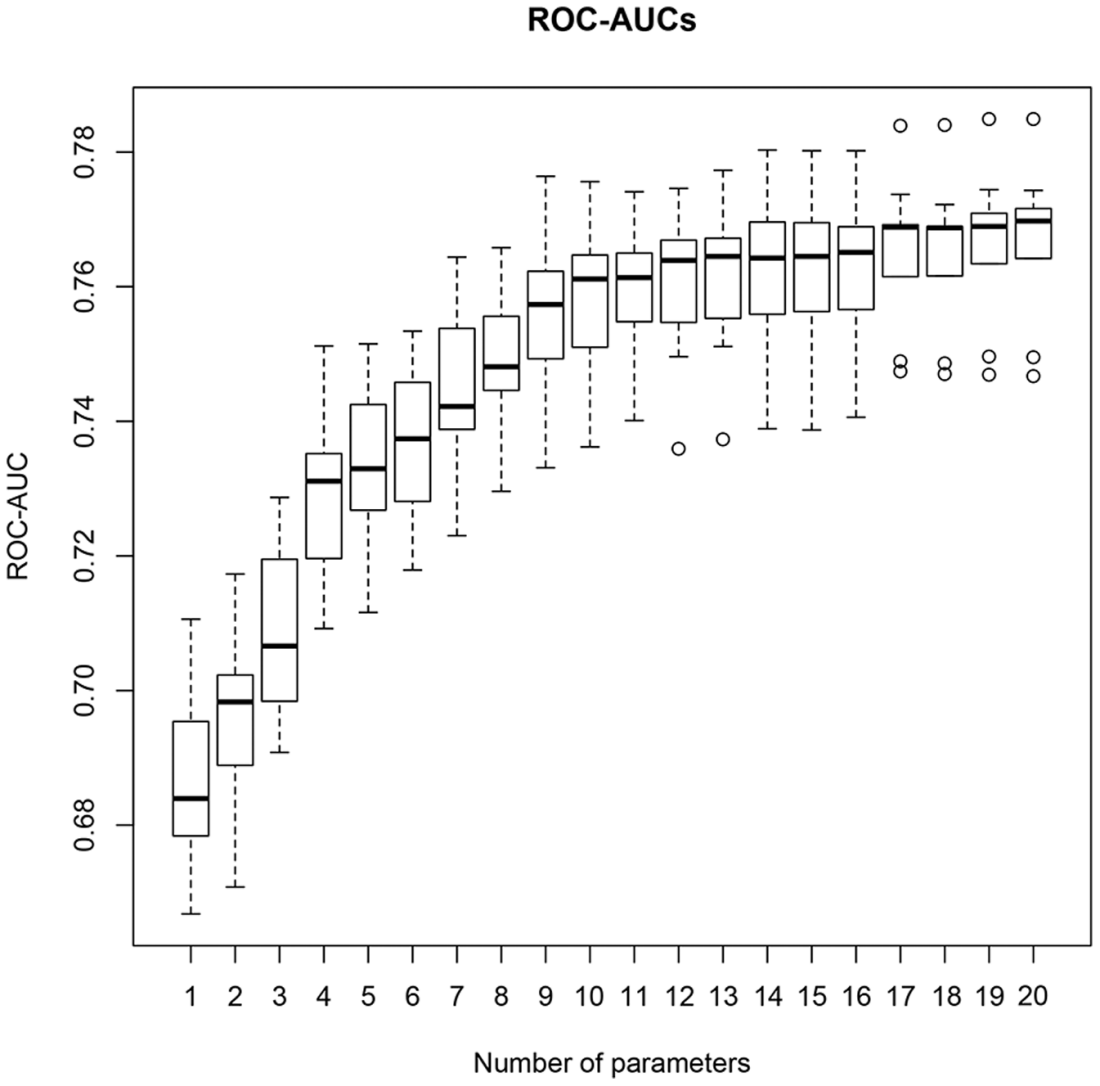
ROC-AUCs assessed in relation to the number of variables used. Variables are ranked according to their individual correlation coefficient with respect to the outcome; significant decrease of the ROC-AUC is seen when more than one variable is deleted.

To estimate the effect of omitting variables with low predictive power, variables of model 1 were ranked according to their individual Pearson correlation coefficient against the outcome variable and deleted step by step in that order. The majority of deletion steps led to a significant decrease of the ROC-AUC. Figure 2 summarizes this deletion procedure.

Due to its retrospective study design, some variables were not available for all patients (Table 2). For most variables less than 10% missing values were observed with the exception of cholesterol (34% missing values), amylase (27%), creatinine kinases (14%) and magnesium (13%). When replacing missing values with the mean value of the corresponding group (“value imputation”), no significant difference in ROC-AUCs were detected (model 1: ROC-AUC = 0.77, p = 0.85; model2: ROC-AUC = 0.76, p = 0.09).

### Validation set

To test the generalizability of the established models, a validation set (n = 1,294) was used. Model 1 achieves an ROC- AUC of 0.80 (CI: 0.76–0.84, see figure S4). Model 2 yields an ROC-AUC of 0.79 (CI: 0.74–0.83). No significant differences were found between ROC-AUCs derived from the validation set and the corresponding ROC-AUCs derived from the derivation set (model 1: p = 0.1542, model 2: p = 0.2594).

**Figure 3:**
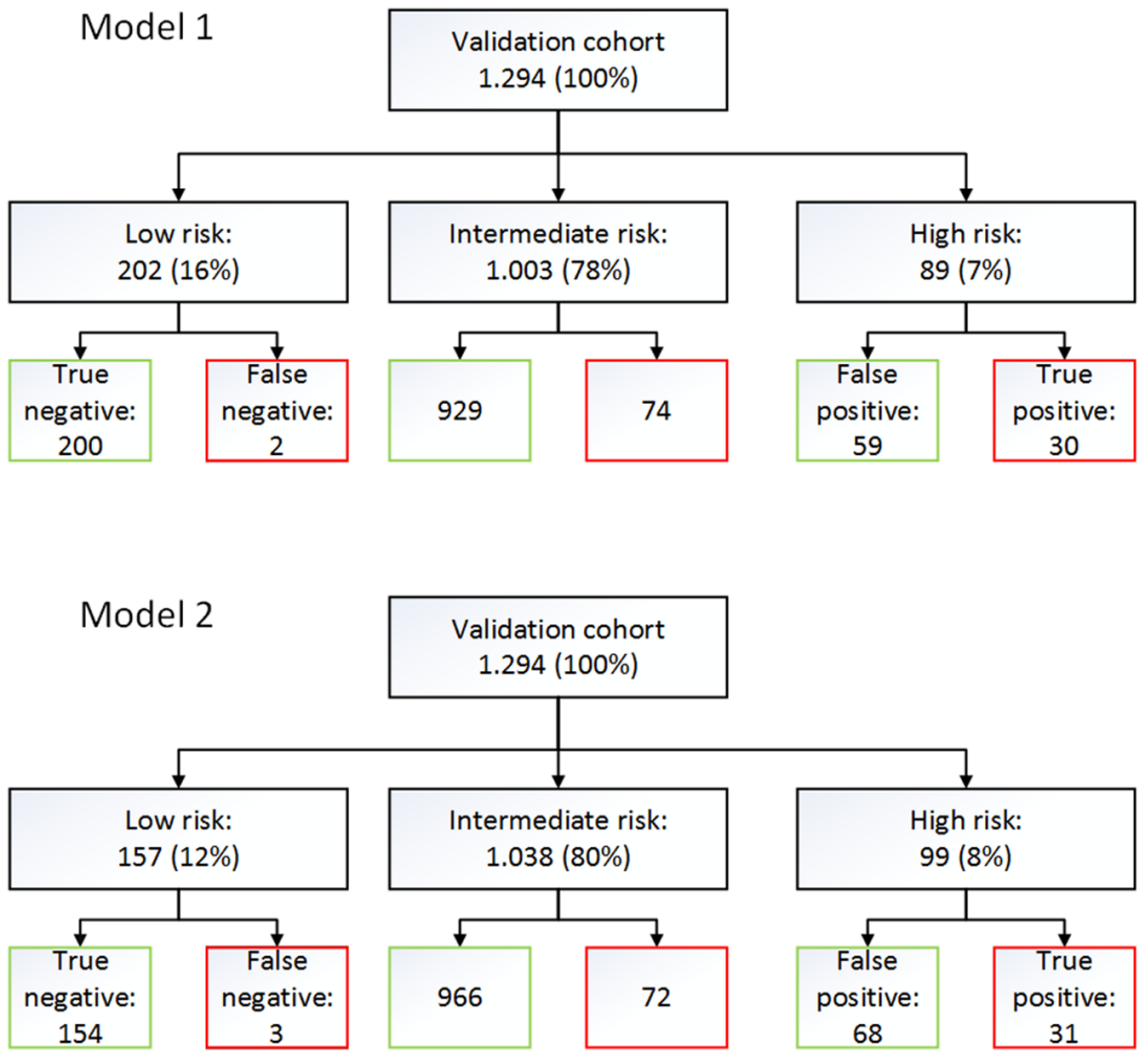
Graphical result of the validation cohort. model 1: 16% low risk cohort with 2 false negative patients; model 2: 12% low risk cohort with 3 false negative patients.

When applying the cut-offs point predefined by the Youden index method in the derivation cohort, model 1 yields a sensitivity of 79.3% and a specificity of 68.4% with 18.4% PPV and 97.4% NPV. Model 2 achieved a sensitivity of 80.2% and a specificity of 70.0% with 19.3% PPV and 97.5% NPV. Using the predefined cut-off points for the risk model, 16% of the patients (n = 202) were allocated to the low risk group and 7% (n = 89) to the high risk group, respectively. Among the patients in the low risk group, only 2 patients were false negatives. Similarly, applying model 2, 157 patients (12%) were allocated to the low risk group with 3 false negatives. Details of the risk model are provided in table 2 while figure 3 represents a tree-based graphical representation of the prediction outcome.

## Discussion

The goal of the current study was to assess the discriminatory power of machine learning models with frequently requested variables for predicting negative blood culture results in inpatients and outpatients with a suspicion to suffer from bacteraemia. The cost effectiveness of blood culture analysis very much depends on the diagnostic yield and therefore an automated tool improving the selection of patients may therefore increase cost-effectiveness. Several scoring systems predicting the probability of a positive blood culture result in a specific patient cohort have been published previously [20], [21], [46]–[48]. However, since these scores necessitate the manual calculation by the physician, these are often not applied. Our approach was to compute a potentially automated decision support tool to improve the cost-effectiveness of blood culture sampling using highly standardized data resulting in ROC-AUCs between 0.759 and 0.804. Based on these models the NPV was 99.01% for model 1 and 98.1% for model 2 for patients of low risk for bacteraemia. Based on these results the proposed support tool would be able to safely reduce 12–16% of blood culture sampling leading to a reduction of costs.

In this study, statistical analysis was restricted to laboratory parameters as well as gender and patient's age, which are all readily available and highly standardized. These variables combine the advantage of reproducibility and availability as opposed to most clinical variables.

Pre-test probability of bacteraemia may vary considerably between studies potentially impacting on the diagnostic accuracy of prediction models [10], [11]. Our results are similar to those of a previous study by Piftenmeyer et al. reporting a 8.2% prevalence of bacteraemia [49]. Nakamura *et. al*. published a hospital based study with a 19.5% prevalence of bacteraemia and predicting bacteraemia with an ROC-AUC of 0.73 [47]. The prevalence of bacteraemia (19.5%) in this study is higher than generally reported for hospital-based studies and may therefore lack generalizability [10], [11]. Finally, Jin *et al*. evaluated a Bayesian algorithm for the prediction of bacteraemia in 19,303 patients, yielding an ROC-AUC of 0.70 [50]. In contrast to our study, however, laboratory markers included in the analysis were allowed a considerable lag time to blood culture sampling of up to 72 hours, or even 7 days in case of albumin and alkaline phosphatise. Considering the dynamic evolution of inflammation makers, this discrepancy in sampling times may have importantly impacted on their results.

Several limitations have to be acknowledged in this study. Firstly, the retrospective nature of the study may introduce bias in the analysis of the results. Although the data set has been split into a sub-set used for model generation and one for validation, the external generalizability needs to be addressed prospectively at other health care institutions. Finally, the applicability of an automated decision support tool needs to be tested in clinical practice. The potential trade-off between diagnostic certainty and economic aspects must be well-balanced and may vary between different settings [51], [52].

In conclusion our data show the utility of highly standardized variables for predicting bacteraemia with an ROC-AUC between 0.759 and 0.800. This prediction model may be tested for implication as clinical support tool to exclude blood culture sampling in patients with very low probability for bacteraemia. A prospective evaluation of the model's generalizability would be indicated.

## Supporting Information

Figure S1 **Fagan's Nomogram.** To graphically represent the correlation between pre-test probability, likelihood ratio and post-test probability; left side: negative likelihood ratio for low risk group cut-off point specification; right side: positive likelihood ratio for high group cut-off point specification.
(TIF)

Figure S2 **ROC-AUCs of various machine learning algorithms.** A: Model 1 (20 variables); resulting in the following ROC-AUCs: A2DE: 0.7671 (CI: 0.754–.781), SVM 0.5 (CI: 0.5–0.5), Naïve Bayes: 0.547 (CI: 0.530–0.563), Multilayer Perceptron: 0.694 (CI: 0.677–0.710), Logistic Regression: 0.751 (CI: 0.737–0.766); B: Model 2 (10 variables), resulting in the following ROC-AUCs: A2DE: 0.759 (CI: 0.745–0.774), SVM: 0.5 (CI: 0.5–0.5), Naïve Bayes: 0.650 (CI: 0.633–0.666), Multilayer Perceptron: 0.729 (CI: 0.714–0.744), Logistic Regression: 0.742 (CI: 0.727–0.757).
(TIF)

Figure S3 **Calibration plots of model 1 and model 2.** x-axis: predicted risk, y-axis: observed risk; a slight overestimation is seen in model 1 for patients with high risk for bacteraemia.
(TIF)

Figure S4 **ROC-AUCs of various the A2DE classifier at the validation set.** Model 1: ROC- AUC: 0.80 (CI: 0.76–0.84). Model 2: ROC-AUC: 0.79 (CI: 0.74–0.83).
(TIF)
