# Supplementary figures and images for: A Risk Prediction Model for Screening Bacteremic Patients: A Cross Sectional Study
Source: PLoS One. 2014 Sep 3;9(9):e106765. doi: 10.1371/journal.pone.0106765 (PMC4153716; doi:10.1371/journal.pone.0106765)

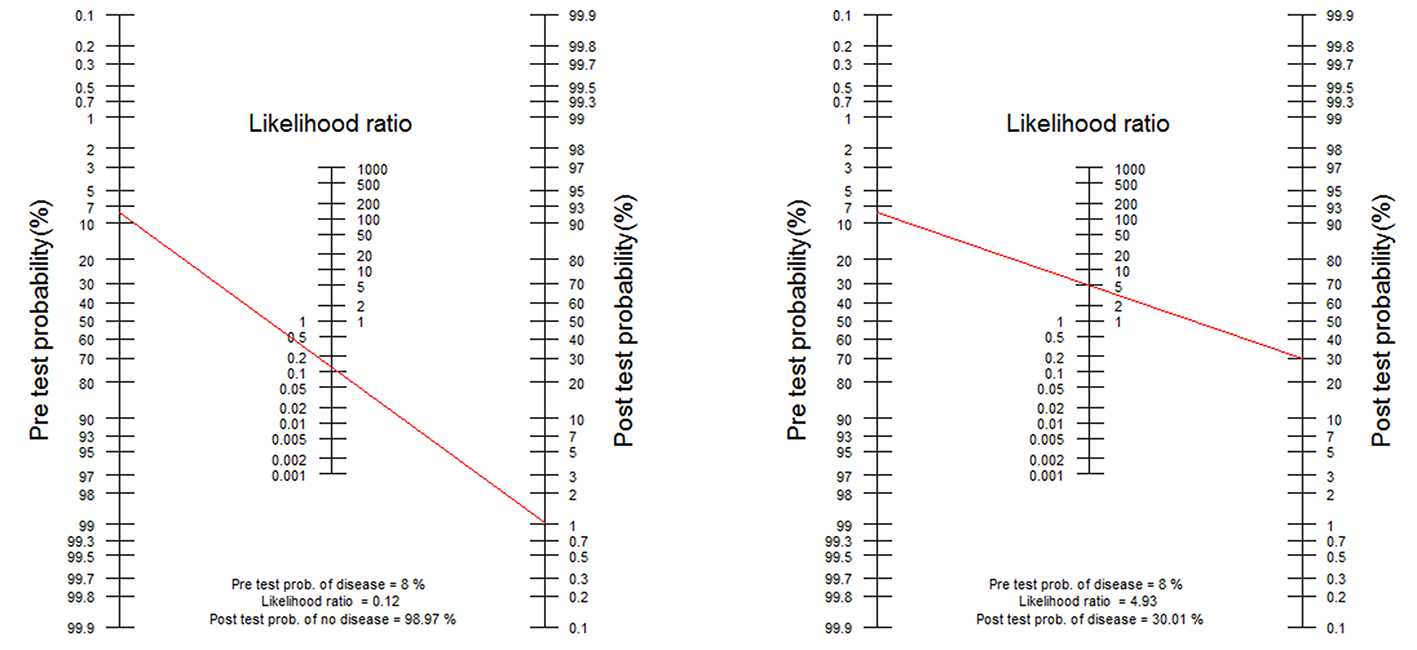

Supplement: Figure S1 — Fagan's Nomogram. To graphically represent the correlation between pre-test probability, likelihood ratio and post-test probability; left side: negative likelihood ratio for low risk group cut-off point specification; right side: positive likelihood ratio for high group cut-off point specification. (TIF) [file pone.0106765.s001.tif]

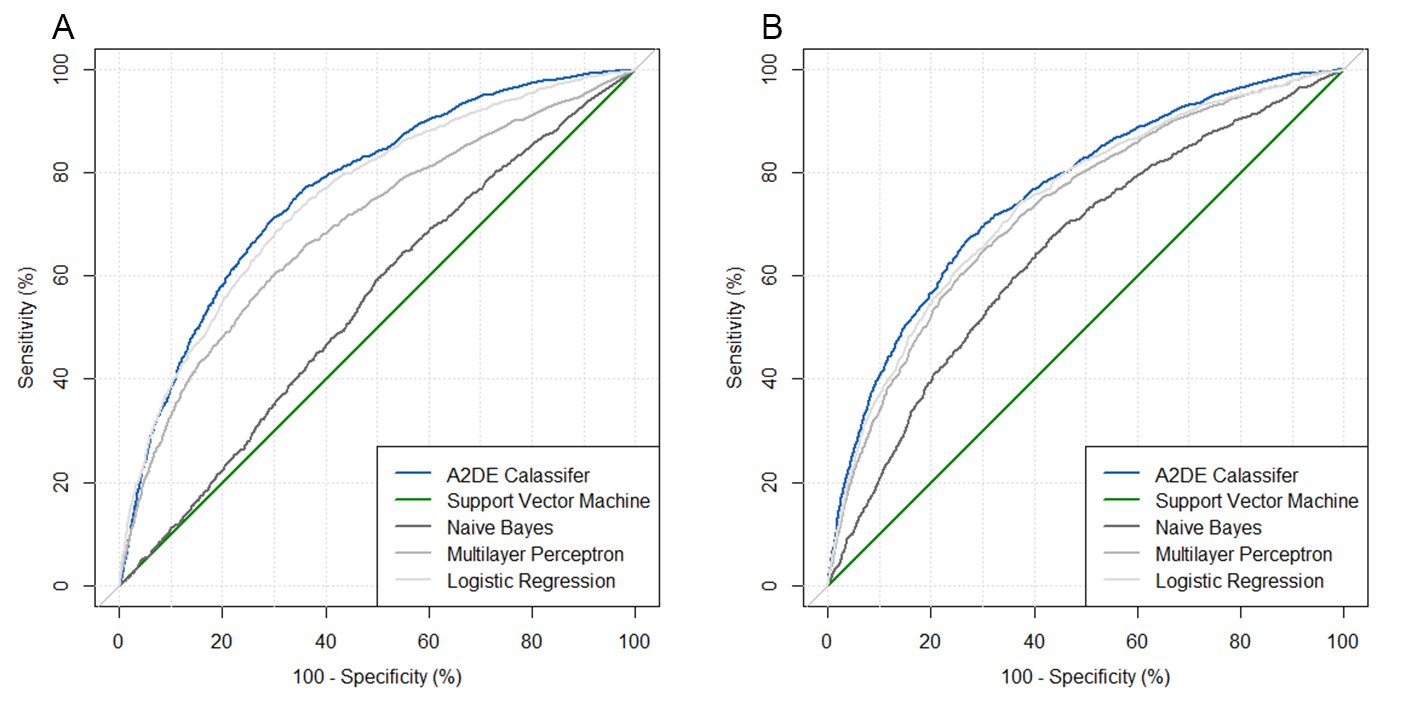

Supplement: Figure S2 — ROC-AUCs of various machine learning algorithms. A: Model 1 (20 variables); resulting in the following ROC-AUCs: A2DE: 0.7671 (CI: 0.754–.781), SVM 0.5 (CI: 0.5–0.5), Naïve Bayes: 0.547 (CI: 0.530–0.563), Multilayer Perceptron: 0.694 (CI: 0.677–0.710), Logistic Regression: 0.751 (CI: 0.737–0.766); B: Model 2 (10 variables), resulting in the following ROC-AUCs: A2DE: 0.759 (CI: 0.745–0.774), SVM: 0.5 (CI: 0.5–0.5), Naïve Bayes: 0.650 (CI: 0.633–0.666), Multilayer Perceptron: 0.729 (CI: 0.714–0.744), Logistic Regression: 0.742 (CI: 0.727–0.757). (TIF) [file pone.0106765.s002.tif]

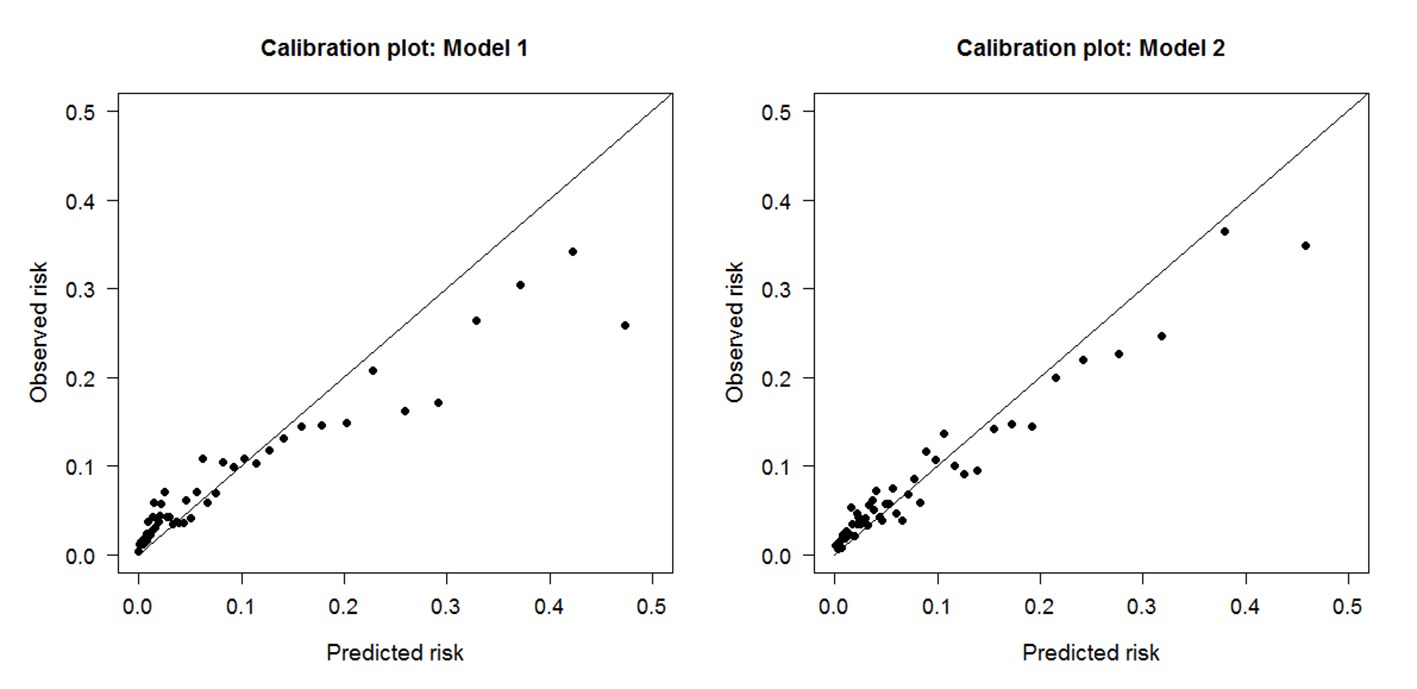

Supplement: Figure S3 — Calibration plots of model 1 and model 2. x-axis: predicted risk, y-axis: observed risk; a slight overestimation is seen in model 1 for patients with high risk for bacteraemia. (TIF) [file pone.0106765.s003.tif]
